# An Effective Meta-analysis of Magnetic Stimulation Therapy for Urinary Incontinence

**DOI:** 10.1038/s41598-019-45330-9

**Published:** 2019-06-24

**Authors:** Qing He, Kaiwen Xiao, Liao Peng, Junyu Lai, Hong Li, Deyi Luo, Kunjie Wang

**Affiliations:** Department of Urology, Institute of Urology (Laboratory of Reconstructive Urology), West China Hospital, Sichuan University, No. 37 Guo Xue Xiang, Chengdu, Sichuan 610041 P.R. China

**Keywords:** Urinary incontinence, Urological manifestations

## Abstract

Magnetic stimulation (MS) is a novel approach for treating urinary incontinence (UI), but its applicability remains unclear. This systematic review and meta-analysis were conducted to evaluate the effects of MS treatment on UI. A literature search was performed in EMBASE, PubMed and Cochrane Library (from May 2018 to August 2018), and all randomized control trials (RCTs) published in English were screened to determine whether they met the inclusion criteria. A manual search of the reference lists of the retrieved studies was also performed. Eleven studies involving 612 patients were included in this review. According to the results of the meta-analysis, MS therapy relieved UI symptoms evaluated using the International Consultation on Incontinence Questionnaire-Short Form (ICIQ-SF) score (mean difference [MD] −3.03, 95% CI −3.27 to −2.79). In addition, the frequency of UI in the MS treatment group was also alleviated compared with sham group (MD −1.42, 95% CI −2.15 to −0.69). Finally, MS treatment improved the quality of life of patients with UI (standardized mean difference [SMD] −1.00, 95% CI −1.24 to −0.76). Our meta-analysis preliminarily indicates that MS treatment is an effective therapeutic modality for patients with UI. Nevertheless, additional large, high quality RCTs with a longer follow-up period that use consistent stimulation methods and analyse comparable outcomes are required to validate the efficacy.

## Introduction

Urinary incontinence (UI), which severely decrease the quality of life (QoL) and sexual function of patients^1^, presents as the complaint of any involuntary loss of urine^2^. Stress urinary incontinence (SUI), which accounts for over half of UI cases, is the symptomatic complaint of involuntary leakage upon effort, exertion, sneezing or coughing^3^. Meanwhile, urgency urinary incontinence (UUI) involves involuntary leakage accompanied by or immediately preceded by a sudden, compelling and uncontrolled desire to pass urine. As the term suggests, mixed urinary incontinence is defined as the combination of SUI and UUI. These three types of UI are the most common forms based on the symptoms, but other types of UI also exist (e.g., continuous UI, nocturnal enuresis, insensible incontinence, and neurogenic UI).

UI is a chronic condition that poses a substantial financial burden on individuals^4^ and society^5^. Most epidemiological studies reported a prevalence of any type of UI ranging from approximately 25% in young adults to 45% in older women^6,7^. A prospective longitudinal study including 1081 urban Swedish women revealed that the overall prevalence of UI increased from 15% in 1991 to 28% in 2007^8^. In this context of an increasing prevalence of UI, susceptible populations require more careful management by health practitioners.

The current initial treatment for all types of UI includes lifestyle interventions, physical therapies, scheduled voiding regimes, behavioural therapies and medication^3^. In particular, pelvic floor muscle training (PFMT) is recommended as a first line therapy for SUI, and PFMT combined with either bladder training or antimuscarinics are advocated for UUI^3^. Other adjunct therapeutic modalities, such as electrical stimulation (ES), vaginal devices and urethral inserts are the second-line options for both patients with SUI and UUI^9^. Magnetic stimulation (MS) treatment is a novel approach to provide noninvasive, passive stimulation to the sacral roots or the pelvic floor. This new form of conservative therapy for UI was approved by the United States Food and Drug Administration in 1998^10^. Pulsed magnetic fields are generated by an electrified coil that induces a flow of ions to form eddy currents when the excitable tissue is exposed to a magnetic field with a sufficient intensity^11^. Therefore, MS depolarizes the motor nerve to produce an action potential that ultimately triggers muscle contractions. Due to the advantages of the lack of an internal probe and requirement for supervision and the ability of magnetic fields to pass through clothing, MS is a very convenient, acceptable and hospitable modality that has attracted increasing attention.

However, the applicability of MS for UI remains unclear^9^. Thus, we conducted this systematic review and meta-analysis to evaluate the effects of MS therapy on UI.

## Results

### Study identification and characteristics

**Figure 1:**
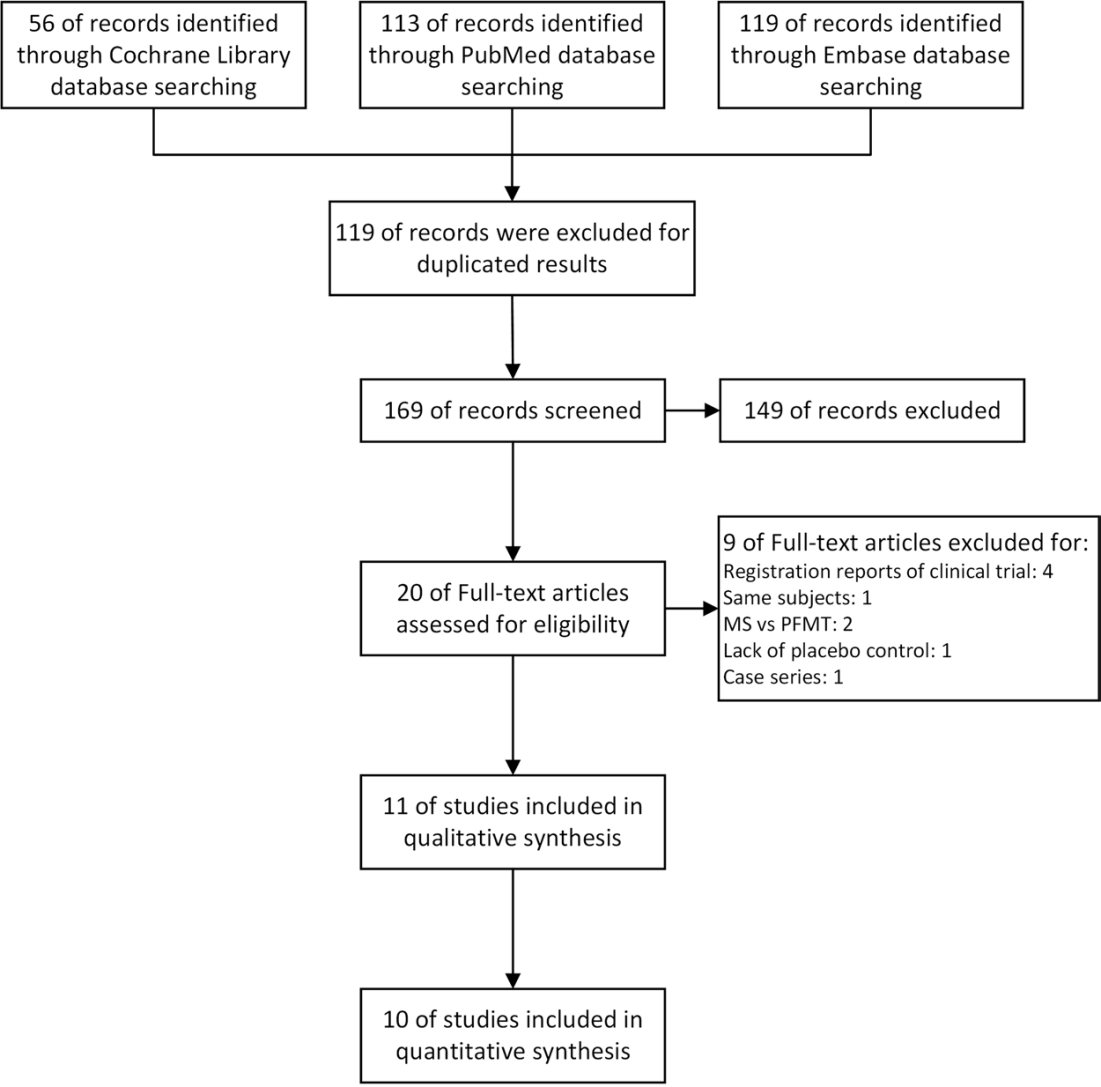
Flow diagram of the systematic review and meta-analysis.

The process used to select the studies included in this article is summarized in Fig. 1. No disagreement occurred between the two reviewers regarding the inclusion of the selected randomized control trials (RCTs). Eleven articles were finally included in this quantitative synthesis (612 patients)^12–22^, and no additional study was identified by searching manual search of the reference lists of these articles. This review identified 3 publications^16,17,23^ that were based on the same RCT^24^. We only extracted unduplicated and useful data from 2 of these studies^16,17^.

Specifically, 6 papers from 5 different RCTs reported outcomes for patients with SUI^12–17^; 3 RCTs compared the effects of MS and a sham device on patients with UUI^18–20^; 1 RCT^21^ focused on patients with MUI and the last RCT^22^ included patients with all types of UI. Overall, 612 participants were enrolled, and 343 patients were assigned to the active MS group and 269 patients were allocated to the sham MS group. Two studies confirmed the type of UI with only a urodynamic examination^14,15^, and 3 studies employed a urodynamic examination and voiding diary to determine the diagnosis^19,21,22^. Five RCTs confirmed the eligibility of the participants based on a voiding diary^12,13,16–18,20^. The detailed characteristics of the selected studies are summarized in Table 1.

**Table 1:** Characteristics of studies included in the meta-analysis.

Study	Jadad score	Type of UI	Diagnosis method	Group	Sample size	Age, mean (SD)	Length of intervention period and frequency	Location	Protocols	Additional therapy	Outcome measures and results	Instrument or questionnaire used	Follow-up period
Fujishiro 2000	0	SUI	Voiding diary	Active	31	58 (37–79)^a^	Only once	Sacral roots	30-min stimulation repetition of 15 Hz in 5-s per minute	None	1. Maximum urethral closure pressure; 2. Frequency of UI; 3. QoL; 4. Objective cure^b^; 5. Improved incontinence^c^	1. Cystometry; 2. 3-day urinary diary; 3. 1-hour pad test	1 week
				Sham	31				Use sham stimulating coil				
Manganotti 2007	0	SUI	Voiding diary	Active	10	50.1 (2.86)	Three sessions per week for 2 weeks	Sacral roots	15-min stimulation repetition cycle of 15 Hz in 3 s per minute	None	1. QoL; 2. Severity of SUI; 3. Stress pad test	1. KHQ; 2. SEAPI-QMM Incontinence Classification System; 3. 1-hour pad test	1 month
				Sham	10				The magnetic coil was positioned over the sacrum in a vertical position				
Gilling 2009	4	SUI	Urodynamic examination	Active	35	54.0 (2.0)	Three treatment sessions per week for 6 weeks	Pelvic floor	1. 10-min stimulation at 10 Hz; 2. 3-min rest; 3. 10-min stimulation at 50 Hz	Low intensity home-based PFMT	1. Frequency of UI; 2. Stress pad test; 3. PFM strength; 4. QoL; 5. ALPP	1. 3-day urinary diary; 2. CVM score; 3. I-QoL; 4. KHQ; 5. Urodynamic test; 6. Perineometer	6-month
				Sham	35	54.8 (2.2)			A thin deflective aluminium plate inserted in the chair				
Yamanishi 2017	4	SUI	Urodynamic examination	Active	18	NA	One session per week for 10 weeks	Pelvic floor	20-min stimulation repetition cycle of 50 Hz in a 5-s “on” 5-s “off” pulsing manner	NA	1. Frequency of UI; 2. Severity of UI; 3. Stress pad test; 4. QoL; 5. ALPP	1. 7-day urinary diary; 2. 24-h pad test; 3. ICIQ-UI SF score; 4. ICIQ-LUTSqol; 5. Urodynamic test	10-week
				Sham	12				1 Hz in 5-s on/5-s off cycles, with a maximum output of ≤42% of the active stimulation				
Lim 2017	5	SUI	Voiding diary, ICIQ-UI SF score	Active	60	51.8 (10.0)	Two sessions per week for 2 months	Pelvic floor and sphincter muscles	20-min stimulation repetition cycle of 50 Hz in an 8-s “on” 4-s “off” pulsing manner	None	1. Frequency of UI; 2. Objective cure^d^; 3. Subjective cure; 4. Stress pad test; 5. PFM function; 6. Severity of UI	1. Urinary diary; 2. ICIQ-UI SF score; 3. Perineometer; 4. PGI-I; 5. ICIQ-LUTSqol; 6. 1-hour pad test	14-month
				Sham	60	52.7 (7.8)			The magnetic coil was tilted 22 degrees down				
Fujishiro 2002	0	UUI	Voiding diary	Active	22	61.3 (8.3)	Only once	Sacral roots	30-min stimulation repetition of 15 Hz in 5-s per minute	None	1. Maximum urethral closure pressure; 2. Frequency of UI; 3. QoL	1. 3-day urinary diary; 2. Cystometry	1 week
				Sham	15	62.7 (8.9)			Use sham stimulating coil				
Suzuki 2007	2	UUI	Urodynamic examination, Voiding diary	Active	20	65.2 (13.1)	One session per week for 10 weeks	Pelvic floor	10 Hz with a pulse width of 300 μs for 20-min	None	1. Frequency of UI; 2. Severity of UI; 3. QoL	1. 7-day urinary diary; 2.ICIQ-UI SF score; 3. ICIQ-LUTSqol; 4. Urodynamic test	24-week
				Sham	19	71.4 (12.6)			1 Hz in 5-s on/5-s off manner with a maximum output of ≤20% of the active stimulation				
Yamanishi 2014	2	UUI	Voiding diary	Active	94	64.1 (13.9)	Two sessions per week for 6 weeks	Pelvic floor	10 Hz with a pulse width of 300 μs for 25-min	None	1. Frequency of UI; 2. Severity of UI; 3. QoL	1. 7-day urinary diary; 2. OABSS; 3. IPSS QoL	None
				Sham	49	67.2 (13.0)			1 Hz in 5-s on/5-s off manner with a maximum output of ≤20% of the active stimulation				
But 2005	3	MUI	Urodynamic examination, voiding diary	Active	23	54.0 (28–70)^a^	Daily use for 2 months	Pelvic floor	18.5 Hz continuous stimulation	None	Severity of UI	Urodynamic test	None
				Sham	16				Inactive stimulation				
But 2003	3	UI	Urodynamic examination, voiding diary	Active	30	55.8 (34–78)^a^	Daily use for 2 months	Pelvic floor	10 Hz with a pulse width of 55 μs for daily	None	1. Frequency of UI; 2. Severity of UI; 3. PFM strength	1. Volume-voided chart; 2. Visual analog scale; 3. Flowmetry; 4. Perineometer	None
				Sham	22				Inactive stimulation				

Abbreviations: UI, urinary incontinence; SUI, stress urinary incontinence; UUI, urgency urinary incontinence; MUI, mixed urinary incontinence; ICIQ-UI SF, Incontinence Questionnaire-Urinary Incontinence Short Form; SD, standard deviation; PFMT, pelvic floor muscle training; NA, not available; QoL, quality of life; PFM, pelvic floor muscle; ALLP, abdominal leak-point pressure; KHQ, King’s Health Questionnaire; CVM, circumvaginal muscle; I-QOL, Urinary Incontinence Quality of Life; ICIQ-LUTSqol, International Consultation on Incontinence Questionnaire-Lower Urinary Tract Symptom Quality of Life; OABSS, overactive bladder symptom score; IPSS, International Prostate Symptom Score. ^a^Mean (range). ^b^No incontinence noted in the voiding diary and leaking of less than 1 gm. ^c^Frequency of incontinence or leaking volume on the pad test decreased by more than 50% compared with the baseline level. ^d^Leakage less than 1 gm on the 1-hour pad test.

**Figure 2:**
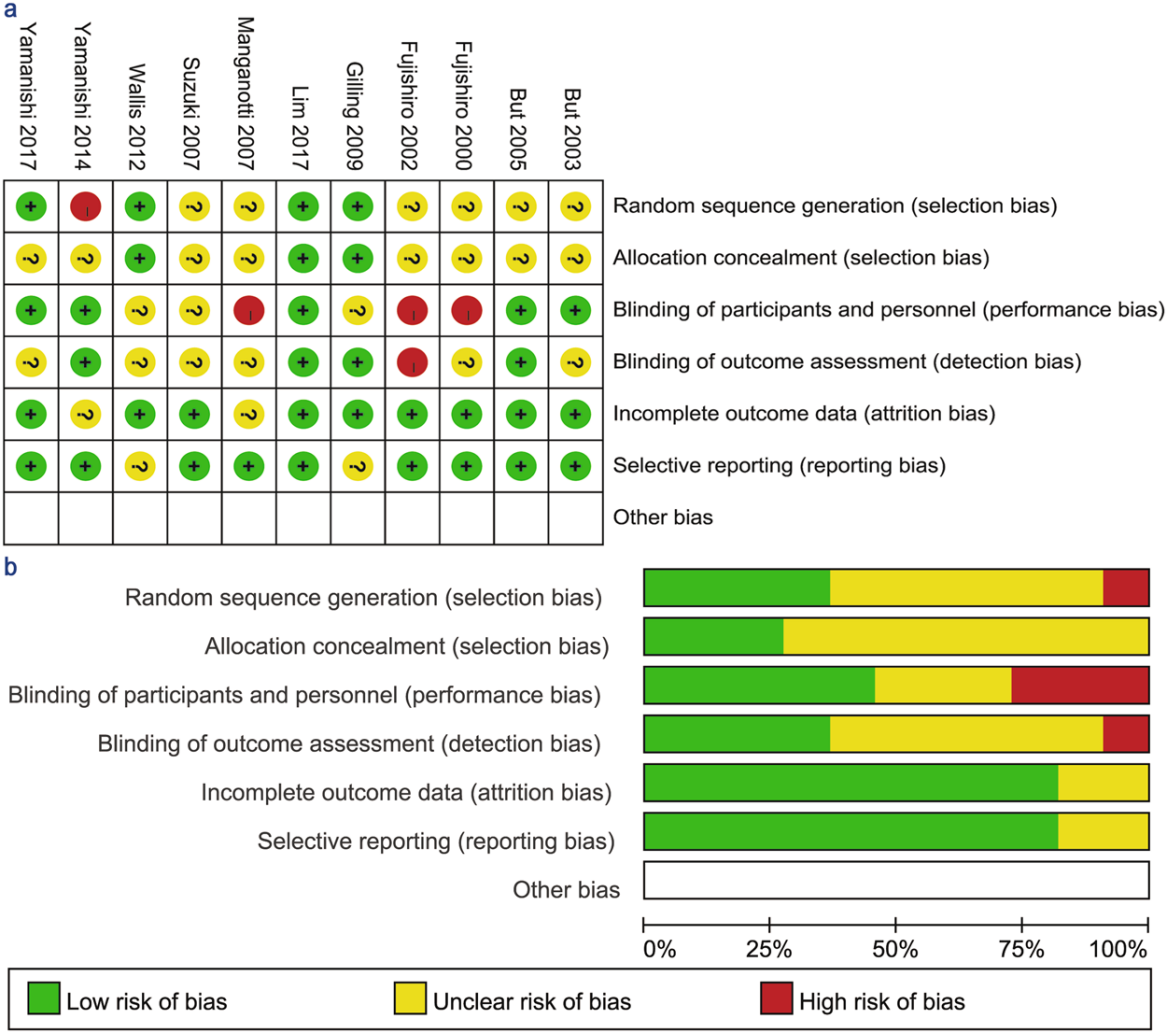
(**a**) Risk of bias summary: review authors’ judgements about each risk of bias item for each included study. (**b**) Risk of bias graph: review authors’ judgements about each risk of bias item presented as percentages for all included studies.

Only 2 RCTs^14,16,17^ reported detailed methods for both random sequence generation and allocation concealment. Six studies^15–17,20–22^ had a low risk of performance bias via blinding of participants and personnel, and the remaining studies had an unclear or relatively high risk of performance bias. Outcome assessments were blinded in 5 studies. In addition, only 2 studies had an unclear risk of attrition bias and 1 article had an unclear risk of reporting bias. The risk of bias graph and summary are illustrated in Fig. 2. Over half of the included studies^14–17,21,22^ were defined as high quality (3–5 points) according to the Jadad scale (Table 1).

Remarkably, significant differences in the frequencies, durations and treatment periods of stimulation were observed, ranging from 10 Hz to 50 Hz, 15 minutes to daily usage, and one single session to 10 weeks, respectively. In addition, the modality used in the sham group was also inconsistent (Table 1).

### Study outcomes

#### UI symptoms

**Figure 3:**
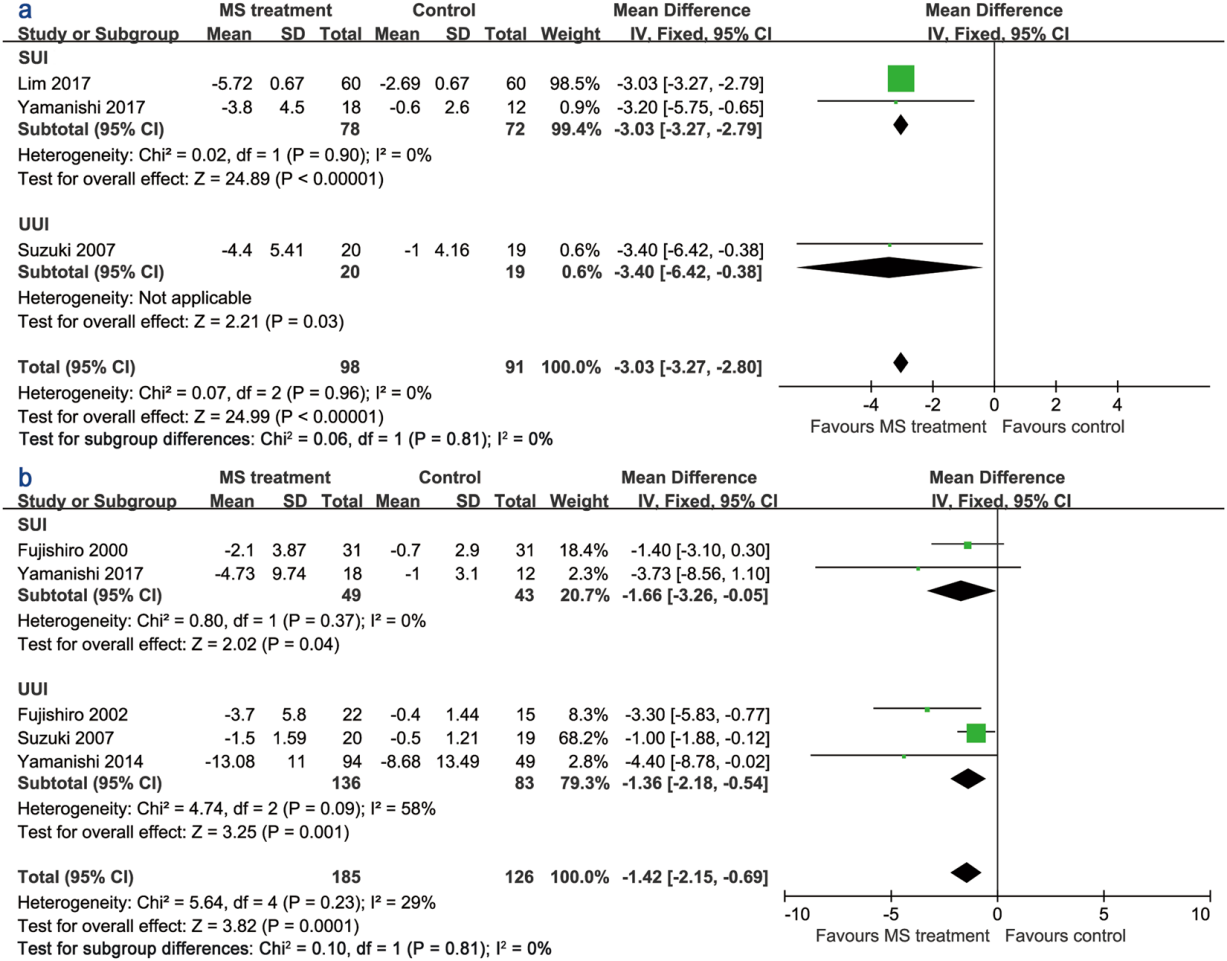
Forest plots comparing the changes in (**a**) the ICIQ-UI SF score and (**b**) UI frequency between the active and sham groups.

Three trials^15,17,19^ provided data about UI symptoms using the International Consultation on Incontinence Questionnaire-Urinary Incontinence Short Form (ICIQ-UI SF) score. High scores on this questionnaire indicated worse symptoms of UI. MS therapy relieved UI symptoms when evaluated using the ICIQ-UI SF score (Fig. 3a). The mean difference (MD) was −3.03 (95% CI −3.27 to −2.79). The treatment period of all studies was at least 2 months. The effect of the MS treatment on UI frequency is illustrated in Fig. 3b. Three studies^15,19,20^ assessed UI frequency with a voiding diary for 1 week, and 2 studies^12,18^ used a diary for 3 days with a short treatment period (single session). The UI frequency of the MS treatment group was alleviated compared with the sham group (MD −1.42, 95% CI −2.15 to −0.69).

#### Cure rates and incontinence improvement

**Figure 4:**
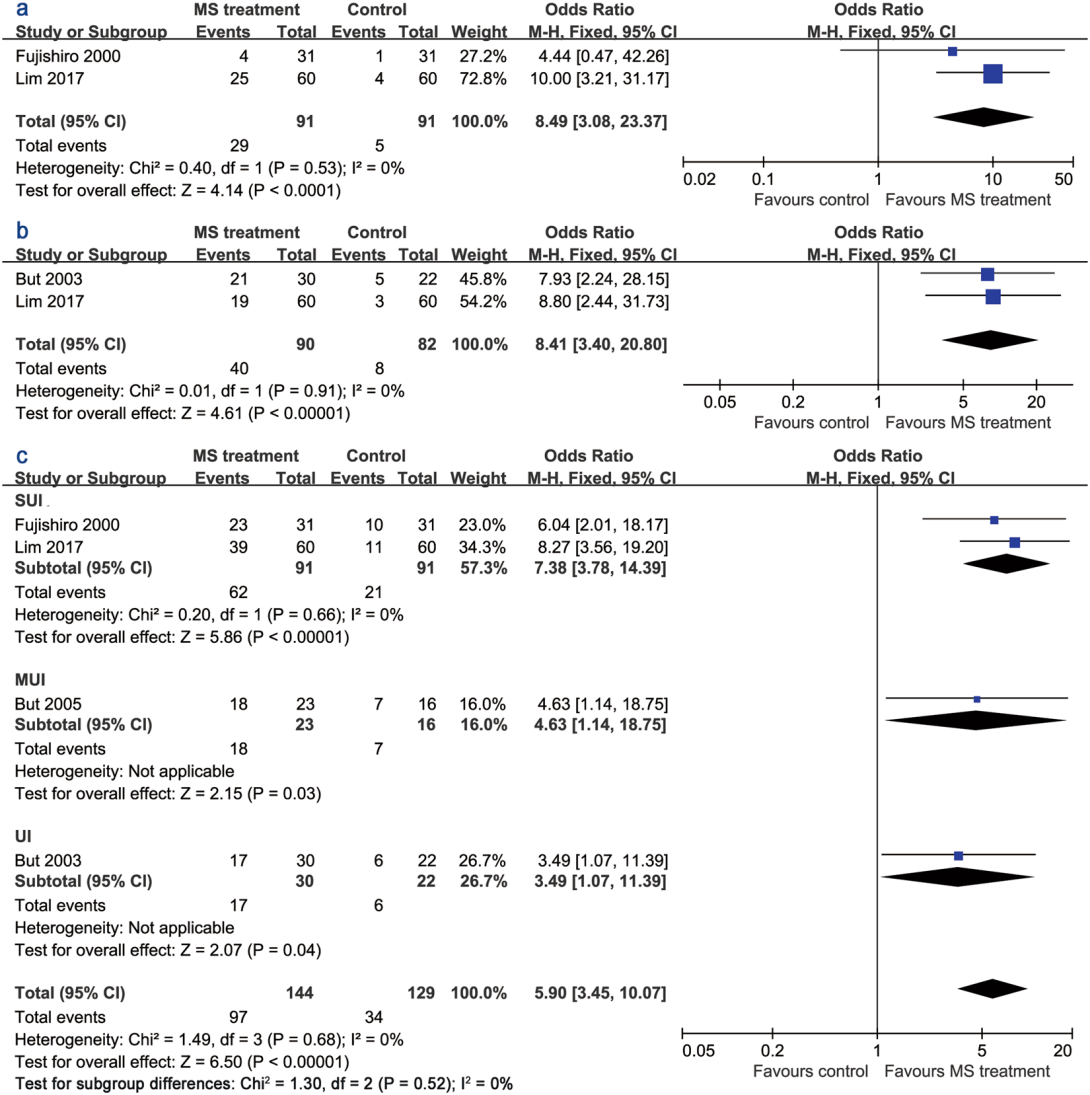
Forest plots comparing (**a**) the objective cure rate, (**b**) subjective cure rate, and (**c**) the incontinence improvement outcome between the active and sham groups.

The pooled data for an objective cure (leakage less than 1 gm on the 1-hour pad test) rate was derived from patients with SUI (Fig. 4a). Patients who received active MS treatment were more likely to be continent when assessed using the pad test (odds ratio [OR] 8.49, 95% CI 3.08 to 23.37). Likewise, the pooled subjective cure rate was significantly higher in the active stimulation group (OR 8.41, 95% CI 3.40 to 20.80) (Fig. 4b). Furthermore, more patients in the MS treatment group exhibited an improvement in incontinence symptoms (OR 5.90, 95% CI 3.45 to 10.07) (Fig. 4c).

#### Stress pad test and urination

**Figure 5:**
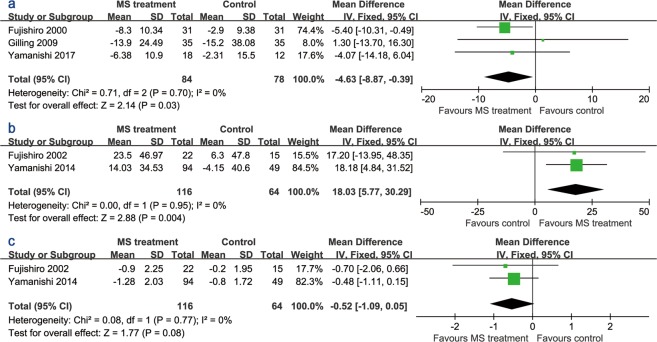
Forest plots comparing the changes in (**a**) the stress pad test, (**b**) mean urine volume per void, and (**c**) micturition number between the active and sham groups.

Three studies of patients with SUI^12,14,15^ provided detailed data from the stress pad test. Compared to the sham group, active stimulation lessened the UI symptoms (MD −4.63, 95% CI −8.87 to −0.39) assessed using the stress pad test (Fig. 5a). However, Fujishiro, *et al*.^12^ used the 1-hour pad test, and other two studies adopted the 24-hour pad test. Active stimulation also improved the process of urination in patients with UUI. As illustrated in Fig. 5b,c, MS treatment increased the mean urine volume per void compared with the sham group (MD 18.03, 95% CI 5.77 to 30.29), although the improvement in the micturition number was not statistically significant (MD −0.52, 95% CI −1.09 to 0.05).

#### QoL score

**Figure 6:**
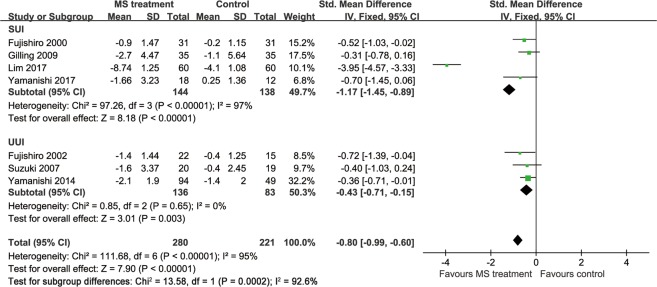
Forest plot comparing the change in the QoL score between the active and sham groups.

**Figure 7:**
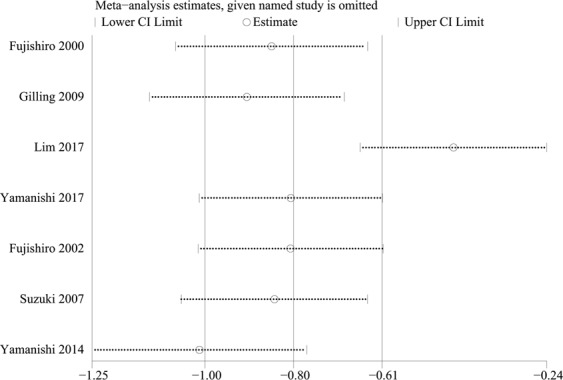
Results of the influence analysis.

**Figure 8:**
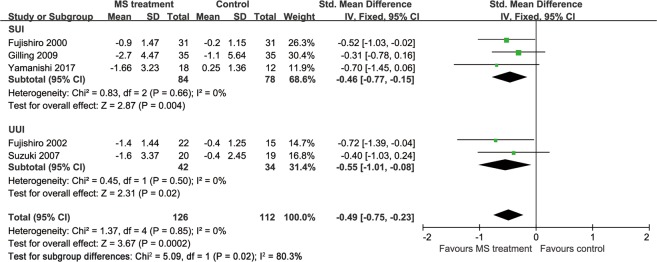
Forest plot comparing the change in the QoL score after omitting studies.

Six studies provided data showing that the MS modality improved the QoL of patients with UI (Fig. 6). Specifically, 4 studies^14,15,17,19^ provided these data using the International Consultation on Incontinence Questionnaire-Lower Urinary Tract Symptom Quality of Life (ICIQ-LUTSqol) score^25^, one study used the International Prostate Symptom Score (IPSS) QoL^20^ and the last 2 studies^12,18^ did not provide adequate information from the questionnaire. High scores on all these questionnaires indicated a poor quality of life. Hence, the MS treatment improved the QoL of patients with UI (standardized mean difference [SMD] −0.80, 95% CI −0.99 to −0.60), but the I^2^ test (95%) revealed heterogeneity. A subsequent influence analysis revealed that the studies conducted by Lim, *et al*.^17^ and Yamanishi, *et al*.^20^ had the greatest influence (Fig. 7). A significant improvement in the QoL was also observed in the active stimulation group (SMD −1.00, 95% CI −1.24 to −0.76) after omitting these studies, and no heterogeneity existed (Fig. 8).

## Discussion

In the present meta-analysis, the UI questionnaire, the frequency of UI, objective and subjective cure rates, the stress pad test, urination condition, and QoL were used to evaluate the efficacy of MS therapy. The active MS treatment decreased UI symptoms (ICIQ-UI SF score, MD −3.03, 95% CI −3.27 to −2.79), alleviated the UI frequency (MD −1.42, 95% CI −2.15 to −0.69), and improved incontinence (OR 5.90, 95% CI 3.45 to 10.07), urination (mean urine volume per void, MD 18.03, 95% CI 5.77 to 30.29) and the QoL of patients with UI (SMD −1.00, 95% CI −1.24 to −0.76) in this meta-analysis.

The ICIQ-SF, an internationally applicable questionnaire, is an effective and validated tool used to quantify the symptoms of UI. Lim, *et al*.^17^, Yamanishi, *et al*.^15^ and Suzuki, *et al*.^19^ all reported that the MS treatment relieved UI symptoms, according to the ICIQ-UI SF score. Recently, a systematic review^26^ including 45 RCTs of surgical or non-surgical interventions for SUI published between January 2015 and July 2017 indicated that the ICIQ-UI SF was used in 18 RCTs, which was the most commonly adopted subjective measurement. More trials using the ICIQ-UI SF will generate more standardized and comparable results in the future, which will strengthen the conclusions of this meta-analysis.

The urinary diary and stress pad test were more objective than the questionnaire in effectively assessing the ability of the MS treatment to alleviate UI symptoms. The reduction in UI frequency was statistically significant in both patients with SUI and UUI (Fig. 3b). The recommended 1-hour stress pad test was conducted as described below. The patient started without voiding and wore a weighed pad. The subject drank 500 ml of a sodium-free liquid within a short period (max. 15 minutes), and then sat or rested 30 minutes. After patient completed a series of physical exercises for 30 minutes, the pad was removed and weighed^27^. Although the pooled data showed a significant improvement in urine loss was significantly improved in active stimulation group, Gilling, *et al*.^14^ and Yamanishi, *et al*.^15^ did not report a significantly greater improvement in the active stimulation group than in the sham group, because they used the 24-hour pad test. Although the ICI recommendation states that pad testing is optional for the routine evaluation of UI and the 24-hour pad test was suggested, several studies have questioned the reliability and reproducibility of the pad test. Simons, *et al*.^28^ assessed the repeatability of the 1-hour pad test in 56 incontinent women and observed significant differences between two tests performed at intervals of 3 to 10 days (MD 9.7 g, 95% CI −66 to 46). Henderson, *et al*.^29^ also concluded that the 24-hour pad test had no significant predictive ability to diagnose SUI. Only less than 10% of urologists routinely perform this test, and several testing protocols with varying recording times exist^30^. Hence, additional studies are required to establish optimal protocols for this test in clinical research and daily care.

In our meta-analysis, the MS treatment led to better objective and subjective cure rates. Additionally, the process of micturition was improved in patients who received active stimulation. Continence is only maintained when the intra-urethral closure pressure exceeds the intravesical pressure both at rest and during periods of increased intra-abdominal pressure^31^. The voiding of urine, which is controlled by reflex mechanisms within the automatic and somatic nervous systems, is also influenced by supraspinal inputs from the central nervous system^32^. As mentioned above, MS is characterized by noninvasive, passive magnetic waves that stimulate the sacral roots or the pelvic floor with the final effect of muscle contraction. For this reason, magnetic stimulation offers an opportunity for patients who may not be motivated to perform regular PFMT for conservative management. Notably, the stimulation is nonspecific, and magnetic waves are not significantly attenuated by the interaction with the tissue. Thus, in addition to the pelvic floor and sacral roots, other muscles, nerves, and even the uterus may react to the stimulation, although most patients tolerate the treatment well.

The QoL is vital for patients with UI, because UI symptoms have been shown to reduce QoL similarly to severe chronic diseases, such as stroke, arthritis and chronic kidney disease^33,34^. An investigation reported an overall prevalence of UI of up to 43.5% in perimenopausal women. Furthermore, few of the affected women sought medical treatment, which in turn had a serious impact on their QoL. In our meta-analysis, patients with UI who received active MS had a better QoL than the sham group. However, 2 studies did not provide detailed information from the QoL questionnaires. The ICIQ-LUTSqol, which is also named King’s Health Questionnaire (KHQ), is a high-quality tool used to assess the impact of LUTS on health-related QoL. Recently, Krhut, *et al*.^35^ included 391 incontinent women and 81 continent volunteers to explore the correlation between incontinence severity and QoL. Even mild urinary leakage significantly reduces the QoL (significantly higher KHQ score). Moreover, a linear correlation between incontinence severity and QoL is not observed.

Our review has several limitations. First, according to the International Consultation on Incontinence and European Association of Urology recommendation, 5 domains of interest should be reported in clinical trials, including patient observations, quantification of symptoms, clinician observations (anatomical, functional, and compliance), QoL, and socioeconomic outcomes^3,9,36^. Unfortunately, only one RCT^24^ planned to report all five domains. Second, the inconsistent protocols for the use of active MS and sham devices, as well as different types of UI, may lead to heterogeneity, although most pooled data were homogeneous. Third, the inconsistent definitions, varying outcome measurements, short length of follow-up, and lack of information about patients who were lost to follow-up may be a potential source of bias. Finally, all included RCTs investigated a relatively small sample size, and data were not sufficient to perform a further analysis such as pelvic floor muscle strength and pressure.

Based on the results of our meta-analysis, MS treatment potentially represents an effective therapeutic modality for patients with UI, as evidenced by the reduced UI symptoms, alleviated UI frequency, increased cure rate, improved micturition, and better QoL. In particular, patients with UI who may not be motivated to conduct regular PFMT can also be treated conservatively with this method. Nevertheless, further large-scale RCTs should be performed to determine consistent intervention protocols and standardize the outcome measurements to generate comparable data. Additionally, a longer follow-up period and a cost-effectiveness analysis will provide more evidence to validate the effects of MS treatment.

## Methods

### Study identification

The present systematic review and meta-analysis was conducted according to the Preferred Reporting Items for Systematic Reviews and Meta-Analyses (PRISMA) checklist^37^. Literature databases, including EMBASE, PubMed and Cochrane library, were searched for all RCTs published in English, and the final search was conducted from May 2018 to August 2018. The Boolean operator “and” was used to combine the search themes. The first theme was magnetic stimulation therapy and expanded versions of the Medical Subject Headings (MeSH) terms *magnetic field therapy* or *electromagnetic therapy*. The last theme was urinary incontinence, combined with the expanded versions of the MeSH terms *stress urinary incontinence* or *urge urinary incontinence*. The publication language was restricted to English.

### Inclusion and exclusion criteria

The potentially relevant articles were independently reviewed by two authors, who reached a consensus on all disagreements. Inclusion criteria were: (1) RCTs evaluating the efficacy of active MS versus sham MS as a treatment for urinary incontinence; (2) study population aged 18 years or older with symptoms of stress urinary incontinence, urgent urinary incontinence or mixed urinary incontinence. Accordingly, studies were excluded based on the following criteria: (1) head-to-head studies of MS versus other modalities (e.g., PFMT and ES); (2) studies including patients who were pregnant; presented with pelvic organ prolapse, severe cardiac/cerebrovascular disorders, urinary tract infection, or a history of pelvic surgery; used medications that may affect urinary incontinence; or received other ongoing treatment for urinary incontinence; (3) abstracts, comments, reviews, conference papers, case reports, meta-analyses and other irrelevant studies. When more than one study included duplicate data from the same population, we only selected the study reporting useful information. Reference lists of selected articles were also examined.

### Data extraction and outcomes

The following data were extracted from each study, if available, using a Microsoft® Excel worksheet: first author’s name, country, year of publication, the number of patients, intervention method, follow-up time, patients’ ages and other characteristics, and the outcomes of urinary incontinence. Dichotomous data were extracted into two-by-two tables. For continuous data, available summary estimates for each group (means and changes in means) and measures of variability (standard deviation [SD]) were extracted. Data were collected by Qing He and the precision of the records was verified by Kaiwen Xiao.

### Evaluation of study quality

Two reviewers independently evaluated the quality of all selected studies, and the final result was recorded after a discussion between these reviewers. The methodological quality of all RCTs was evaluated using the Jadad score^38^ and Cochrane risk of bias assessment tool.

### Statistical analysis

We used RevMan version 5.3 software (Cochrane Collaboration, Oxford, UK) to perform the meta-analysis. The efficacy of the MS treatment was assessed by calculating the OR, MD and SMD, along with the corresponding 95% confidence interval (CI), for the comparison between active stimulation and sham stimulation. The pooled value was calculated using the Z test. In addition, if *p* < 0.05, the difference was considered statistically significant. The heterogeneity among studies was evaluated using the Cochrane Q statistic (significance level of *p* ≤ 0.10) and the inconsistency (I^2^) test. If heterogeneity was observed, the influence analysis (Stata 15.0, Stata Corp, College Station, Texas) was used to identify the study with the greatest influence on the pooled data. If heterogeneity still existed after omitting this study, the random effects model was used to generate the most conservative estimate.
